# The Relationship between NAFLD and Sarcopenia in Elderly Patients

**DOI:** 10.1155/2018/5016091

**Published:** 2018-03-26

**Authors:** Yu Zhai, Qian Xiao, Jing Miao

**Affiliations:** ^1^Department of Geriatrics, The First Affiliated Hospital of Chongqing Medical University, Chongqing 400016, China; ^2^Department of Public Health College, Chongqing Medical University, Chongqing 400016, China

## Abstract

**Aim:**

Previous studies have shown that individuals with low muscle mass exhibit an increased risk of nonalcoholic fatty liver disease (NAFLD). In this study, we investigated the association between NAFLD and sarcopenia in elderly patients.

**Methods:**

We classified the participants into sarcopenia/nonsarcopenia groups based on dual-energy X-ray absorptiometry (DXA), muscle strength (grip strength), or/and physical performance (6 m usual gait speed). We diagnosed NAFLD by ultrasonography combined with the history of alcohol intake. Logistic regression analysis was used to assess the correlation between sarcopenia and NAFLD.

**Results:**

NAFLD was significantly less frequent in the sarcopenia group than in the nonsarcopenia group (*P* < 0.01). However, NAFLD was neither an independent risk factor nor a protective factor for sarcopenia.

**Conclusions:**

NAFLD is not independently associated with sarcopenia.

## 1. Introduction

Many challenges have emerged from the aging of society, and several growing health problems related to aging, including sarcopenia, need to be addressed by geriatric researchers. The term sarcopenia is derived from the Greek word for the loss of flesh and was first suggested by Rosenberg in 1989 [1]. Owing to an increasing number of basic and clinical studies, the definition of sarcopenia has been refined. In 2010, the European Working Group on Sarcopenia in Older People (EWGSOP) reported sarcopenia as “a syndrome characterized by progressive and generalized loss of skeletal muscle mass and strength with a risk of adverse outcomes such as physical disability, poor quality of life and death” [2].

Nonalcoholic fatty liver disease (NAFLD) is a genetic environment-associated metabolic stress-related disease, and it has replaced viral liver diseases as the most common liver disease around the world. For example, the Third National Health and Nutrition Examination Survey in the United States showed that the prevalence rate of NAFLD was 19.0% [3]. Recent studies show that NAFLD and sarcopenia share common pathological and physiological mechanisms; furthermore, skeletal muscle mass index (SMI) and hepatic steatosis are negatively correlated [4, 5].

In this study, we focused on NAFLD in elderly patients. According to the latest criterion, sarcopenia is diagnosed through a combination of muscle mass index, grip strength, and 6 m usual gait speed. The correlation between sarcopenia and NAFLD was discussed through a cross-sectional study. Similar studies had not been performed until now.

## 2. Materials and Methods

*Materials.* The subjects signed informed consent approved by the ethics committee of the First Affiliated Hospital of Chongqing Medical University. From June 2014 to June 2017, in the First Affiliated Hospital of Chongqing Medical University, inpatients older than 60 years at the geriatric department and endocrinology department, who had a stable condition, were included in the study. Patients with New York Heart Association (NYHA) class III disease, acute exacerbation of chronic obstructive pulmonary disease (AECOPD), and acute stroke, among others, were excluded. Patients who had renal replacement therapy, stage 5 chronic kidney disease (estimated glomerular filtration rate < 15 * *mL/min), history of organ transplant, extrahepatic fibrosis, or secondary causes of fatty liver or patients who had taken immunomodulators within the preceding 6 months were also excluded. Dual-energy X-ray absorptiometry (DXA) and abdominal ultrasound were performed under the observation of routine physicians. Tests for 6 m usual gait speed were also performed. According to the recommendations of American Society of Hand Therapists (ASHT) [6], the left and right hands were each measured with Jamar digital hand dynamometer three times, and the maximum value was used.

### 2.1. Clinical and Laboratory Measurements

The patients who had completed DXA, tests for upper grip strength, and 6 m walking speed were selected. According to the recommendations of the Asian Working Group for Sarcopenia by using cutoff values for appendicular skeletal muscle mass/height^2^ (7.0 kg/m^2^ for men and 5.4 kg/m^2^ for women by DXA), handgrip strength (<26 kg for men and <18 kg for women), and usual gait speed (<0.8 m/s), we divided the patients into a sarcopenia group and a nonsarcopenia group. According to the Fatty Liver and Alcoholic Liver Disease Study Group of Chinese Liver Disease Association along with the World Gastroenterology Organization Global Guidelines [7], the clinical and imaging diagnosis of NAFLD included the exclusion of significant alcohol consumption (≥20 g/d) and demonstration of hepatic steatosis by liver ultrasound in the presence of metabolic risk factors and other causes of hepatic steatosis or other chronic liver diseases. 

*Other Materials*. After the patients had fasted for 8~12 h, their height, weight, and seated blood pressure were measured, and peripheral venous blood was obtained and analyzed using an automatic biochemistry analyzer to measure the levels of blood uric acid, alanine, aminotransferase (ALT), aspartate aminotransferase (AST), hypersensitive C-reactive protein (hs-CRP) and glycosylated hemoglobin (HbA1c), creatinine, total cholesterol (TC), triglyceride (TG), low density lipoprotein (LDL), and high-density lipoprotein (HDL). Physicians collected medical history. Additionally, body mass index (BMI) was calculated according to the following formula: BMI = weight (kg)/height (m)^2^.

### 2.2. Statistical Analysis

The software Statistical Analysis System  8.0 was used for all data analysis. Continuous variables are represented by the mean ± standard deviation. Chi-square test was used for categorical variables, and Student's *t*-test was used for continuous variables to assess statistical significance of differences between two groups. Logistic regression analysis was used to assess the correlation between sarcopenia and NAFLD, HbA1c, hs-CRP, BMI, age, and sex. *P* < 0.05 was considered statistically significant.

## 3. Results

A total of 494 patients aged 60 to 96 years were enrolled in this study (216 males and 278 females). The average age of the patients was 71.28 years. There were 158 cases (87 males and 71 females) in the sarcopenia group and 336 cases (129 males and 207 females) in the nonsarcopenia group. The average age and excess hs-CRP rate in the sarcopenia group were higher than those in the nonsarcopenia group, while the average BMI and the incidence of NAFLD were lower than those in the nonsarcopenia group (*P* < 0.05, Table 1). Sarcopenia and NAFLD were negatively correlated (Table 2).

In the logistic regression analysis, BMI, sex, age, high blood pressure, diabetes, HbA1c, high uric acid hematic disease, hs-CRP, levels of ALT, AST, TC, TG, LDL, and HDL, and NAFLD were independent variables, and sarcopenia was the dependent variable. Additionally, BMI was a protective factor for sarcopenia, while age, sex, and hs-CRP were risk factors for sarcopenia (*P* < 0.05, Table 3). NAFLD was neither a risk factor nor a protective factor for sarcopenia.

## 4. Discussion

Sarcopenia is characterized by reduced muscle mass and decreased muscle function, both of which increase the risk of falls and reduce the ability of elderly individuals to live on their own. The rising prevalence of NAFLD has paralleled the obesity epidemic in western and developing countries, which will soon make NAFLD the most common liver disease worldwide [8]. Sarcopenia has been recognized as a new geriatric syndrome. The risk of NAFLD increases with age [9]. With increasing age, both reduced muscle mass and presence of NAFLD threaten the health of elderly populations. Studies have explored the existence of an intrinsic correlation between the two conditions, and muscle loss was identified as a risk for NAFLD [10, 11]. NAFLD can be clearly diagnosed, combined with alcohol intake history, and ultrasound manifestation with good sensitivity and specificity. In this study, abdominal ultrasound was used to diagnose NAFLD.

In our study, the results of logistic multiple regression analysis showed that age, hs-CRP, sex, and BMI were associated with sarcopenia. Age is a risk factor for sarcopenia. The risk of suffering from sarcopenia in the oldest age group (≥80 years) is 3.504 times higher than that in younger age groups (<70 years) (odds ratio (OR) = 3.504; *P* < 0.05). At present, it is believed that chronic inflammation is one of the pathophysiological mechanisms underlying sarcopenia. Excess hs-CRP, with a 2.283 times higher risk of sarcopenia than normal values (OR = 2.283; *P* < 0.05), is also a risk factor for sarcopenia. In our study, men were 2.417 times more likely to suffer from sarcopenia than were women (OR = 2.417; *P* < 0.05). Androgens may play an important role in maintaining muscle mass. The decrease in androgen levels in men with increasing age may lead to an increased prevalence of sarcopenia. In addition, elderly women participate more in physical activity, such as housework, shopping, and square dancing, in their daily life than do men. It is known that exercise increases muscle mass. We also found that the incidence of sarcopenia decreased with increasing BMI. Compared with the group with BMI < 18.5 kg/m^2^, there was a significant decrease in the risk of sarcopenia in the group with BMI ≥ 24 kg/m^2^ (OR = 0.0342; *P* < 0.05). Muscle is one of the important components of body mass; thus, when muscle mass increases, BMI does as well. Therefore, BMI is a protective factor to sarcopenia.

Our conclusions are different from the Korean Sarcopenic Obesity Study [11]. Correlation analysis between NAFLD and sarcopenia showed that the two diseases are negatively correlated (*R* = −0.15; *P* = 0.001). NAFLD is neither a risk factor nor a protective factor for sarcopenia according to the results of our logistic multiple regression analysis. Additionally, the Korean study lacked measurement of muscle strength and muscle function and was not specifically designed for the elderly. The study showed that muscle loss was a risk factor to NAFLD, but it did not demonstrate the relationship between sarcopenia and NAFLD. Low muscle mass and high fatty mass arise simultaneously in sarcopenic obesity. However, the prevalence of sarcopenic obesity in the elderly is approximately 5.8% [12]. In conclusion, previous studies on the relationship of sarcopenia and NAFLD need to be revised.

Nevertheless, the present cross-sectional study has some limitations. First, the selection of subjects may be affected by certain sampling errors. Second, it is possible that the coexistence of multiple diseases and long hospitalization may have influenced the final result. Therefore, the exact link between NAFLD and sarcopenia remains to be explored further.

## Figures and Tables

**Table 1:** Clinical characteristics of the study population.

	Sarcopenia	Nonsarcopenia
Number	158	336
Male/female	87/71	129/207
Hyperuricemia (%)	18.99%	20.24%
Diabetes (%)	74.05%	80.4%
Hypertension (%)	56.32%	56.55%
ALT (U/L)	22.85 ± 21.42	22.08 ± 16.77
AST (U/L)	23.14 ± 14.71	22.16 ± 15.44
HbA1c	7.79 ± 2.33	8.2 ± 2.36
TC (mmol/L)	4.38 ± 2.80	4.32 ± 1.53
TG (mmol/L)	1.50 ± 1.83	1.71 ± 1.65
HDL (mmol/L)	1.26 ± 0.39	1.21 ± 0.38
LDL (mmol/L)	2.47 ± 0.90	2.58 ± 0.97
NAFLD (%)	22.15%^*∗*^	37.20%
Excess hs-CRP (%)	48.10%^*∗*^	30.95%
Age (years)	73.75 ± 8.52^*∗*^	70.12 ± 6.95
BMI (kg/m^2^)	22.13 ± 2.97^*∗*^	25.02 ± 3.34

^*∗*^ *P* < 0.05.

**Table 2:** Correlation coefficients between sarcopenia and NAFLD.

	NAFLD	
	*R*	*P*
Sarcopenia	−0.15	0.001

**Table 3:** Logistic regression analysis of the association between sarcopenia and NAFLD, BMI, age, sex, and hs-CRP.

	Sarcopenia	OR	95% CI
BMI (<18.5)	78.26%	Control	
BMI (~18.5)	45.50%	0.185	0.123–0.279
BMI (≥24)	15.66%	0.0342	0.0151–0.0778

Age (<70)	25.79%	Control	
Age (~70)	28.57%	1.872	1.394–2.512
Age (≥80)	55.95%	3.504	1.943–6.310

Female	35.97%	Control	
Male	40.28%	2.417	1.553–3.762

hs-CRP (normal)	26.11%	Control	
hs-CRP (excess)	42.2%	2.283	1.459–3.573

*P* < 0.05.
